# Cognitive function, anxiety, and depression in patients newly diagnosed with thyroid cancer in China

**DOI:** 10.3389/fpsyg.2026.1908212

**Published:** 2026-09-21

**Authors:** TaiXu Jiang, HongShi Shi, YueNan Zheng, Pai Liu, MoFei Wang

**Affiliations:** 1 Department of Thyroid, Breast and Hernia Surgery, Affiliated Hospital of Inner Mongolia Minzu University, Tongliao, Inner Mongolia, China; 2 Department of Thyroid Surgery, The First Hospital of China Medical University, Shenyang, Liaoning, China

**Keywords:** anxiety, cognitive dysfunction, depression, differentiated thyroid cancer, newly diagnosed patients

## Abstract

**Background:**

Despite excellent prognosis, cognitive and emotional disturbances have been reported in survivors of differentiated thyroid cancer (DTC). However, few studies in China have described baseline cognitive and emotional status in patients with newly diagnosed DTC prior to treatment.

**Objective:**

To investigate possible cognitive impairment as well as anxiety and depression levels in patients newly diagnosed with DTC before treatment, and to explore their associated factors, so as to provide baseline data regarding cognitive and emotional disturbances among Chinese patients with DTC.

**Methods:**

This cross-sectional study enrolled patients with DTC treated at a tertiary referral hospital between July and September 2023. Participants completed the Montreal Cognitive Assessment (MoCA), Self-Rating Anxiety Scale (SAS), Self-Rating Depression Scale (SDS). Chi-square tests were used for intergroup comparisons, while univariate and multivariate logistic regression analyses were performed to explore factors associated with possible cognitive impairment, anxiety, and depression.

**Results:**

A total of 708 preoperative patients with newly diagnosed DTC were enrolled. Among them, 7.2% presented anxiety symptoms, 20.3% presented depression symptoms, and 62.7% exhibited possible cognitive impairment. Among female participants, postmenopausal status was associated with possible cognitive impairment. Multivariate logistic regression showed that age (*p* = 0.003) and educational levels (*p* < 0.05) were independent correlates of possible cognitive impairment among women, while age (*p* < 0.001), work status (*p* < 0.05) and residence (*p* = 0.007) were independently correlated with possible cognitive impairment in the overall cohort. Elevated TSH was a correlate of anxiety (*p* = 0.014). Income status was independently associated with depressive symptoms (*p* < 0.05).

**Conclusion:**

Psychological and cognitive disturbances are common among preoperative with DTC. Age, postmenopausal status, educational levels, work status, and residence are related to possible cognitive impairment. TSH levels are relevant correlates of anxiety, and income status is correlated with depression. Routine psychological and cognitive screening should be considered for preoperative DTC patients.

## Introduction

Thyroid cancer is one of the most common endocrine malignancies and its incidence is steadily increasing yearly (Lin et al., 2021). Despite the excellent prognosis of most thyroid cancers, patients still suffer from disturbances across multiple cognitive domains, including executive function, psychomotor speed, and attention (Jaracz et al., 2012), alongside emotional disorders such as anxiety and depression (Dionisi-Vici et al., 2021). These symptoms significantly affect patients’ quality of life and disease-related prognosis (Larrabee et al., 2023). Previous reports have indicated that the quality of life of differentiated thyroid cancer (DTC) survivors is markedly lower than that of the general population and that of other cancer types with poorer prognoses (van de Wal et al., 2016).

Multiple studies have reported mental disturbances in DTC patients, yet most of these investigations have concentrated on postoperative populations (Gou et al., 2017; Gülsoy Kirnap et al., 2020; Jin et al., 2019; Jung and Visovatti, 2017; Qiao et al., 2022; Su et al., 2023). Compared with healthy controls, DTC patients demonstrate deficits in attention and working memory function (Jung and Visovatti, 2017). Thyroid stimulating hormone (TSH) suppression therapy effectively reduces tumor recurrence and metastasis. Even so, patients receiving this treatment often experience anxiety and depressive symptoms, accompanied by significant changes in short-term memory, attention, and word selection ability across different treatment stages (Gülsoy Kirnap et al., 2020; Jin et al., 2019). Although radioactive iodine (RAI) therapy represents an important treatment for DTC, many patients develop notable anxiety and depression before and after RAI administration. This psychological burden is more evident among female patients with lower education levels and household income (Qiao et al., 2022; Su et al., 2023), reducing their overall quality of life (Gou et al., 2017).

To our knowledge, only one relevant Korean study has assessed pretreatment cognitive function among female DTC patients. In that report, up to 95% of participants experienced cognitive impairment, 13.7% had depressive symptoms, and 58.9% experienced varying degrees of psychological distress (Jung et al., 2022). Nevertheless, that work was limited by its small sample size, exclusive female enrollment, and a lack of adequate predictive indicators. Importantly, to date no investigations have specifically addressed possible cognitive impairment, anxiety, and depression among newly diagnosed patients with DTC in China, pointing to an important unaddressed research gap.

Therefore, the aim of this large-scale study was to determine the prevalence of possible cognitive impairment, as well as anxiety and depressive symptom levels, among Chinese patients with DTC prior to treatment. We further explored the factors associated with these cognitive and emotional abnormalities to provide baseline data on potential cognitive impairment and emotional disorders, and to lay a foundation for investigating their dynamic changes during disease progression.

## Materials and methods

### Patients and study design

This cross-sectional study included patients who were scheduled for thyroid surgery at The First Hospital of China Medical University between July and September 2023. Patients were asked to fill out a self-report questionnaire before surgery, and two psychologists conducted neuropsychological assessments in a quiet setting.

Patients who provided written informed consent and who were scheduled for treatment within 3 months after pathological DTC confirmation were enrolled in this study. The exclusion criteria included the following: (1) age <18 years; (2) a history of thyroid surgery; (3) postoperative pathological confirmation not of DTC; (4) a history of anxiety, depression, or regular use of psychotropic medications, including antidepressants, antipsychotics, sedative-hypnotics, mood stabilizers, and anxiolytics; (5) impaired listening, speaking, reading, or writing ability; (6) refusal to cooperate; (7) incomplete clinical data. Incomplete clinical data were defined as missing questionnaire-scale information or core demographic data (e.g., age, work status, and income status). Partially missing thyroid function parameters did not result in study exclusion. Baseline demographic and clinicopathological data were collected, including gender, age, body mass index (BMI), comorbid conditions, educational levels, marital status, work status, income status, residence, preoperative fine-needle aspiration (FNA) results, and thyroid function parameters (FT3, FT4, and TSH). Comorbid conditions were defined as pre-existing chronic illnesses prior to thyroid surgery, including hypertension, diabetes mellitus, and coronary heart disease. Among female patients, postmenopausal status was defined as 12 consecutive months of spontaneous amenorrhea without pathological causes. Women who did not meet this criterion were considered premenopausal.

### Instruments

In 1971, Zung compiled the Self Rating Anxiety Scale (SAS) to assess anxiety level (Dunstan and Scott, 2020), and in 1965, he compiled the Self Rating Depression Scale (SDS) to assess depressive status (Zung et al., 1965). The Montreal Cognitive Assessment Scale (MoCA) was used to assess seven cognitive function domains: visual space and executive function, naming, attention, language, abstraction, delayed recall, and directional ability (Nasreddine et al., 2005). These instruments have good sensitivity and reliability and are widely used in clinical practice. Detailed scoring rules and cutoff values are shown in Table 1.

**Table 1:** Psychological questionnaire and scoring principles.

Test/measure	Neuropsychological domain	Scoring methodology	Scoring principles
MoCA	Visuospatial‑executive function	Total score = 30 points.1 point is awarded for participants with ≤12 years of education.	
	Naming		
	Attention		
	Language		<26: Possible cognitive impairment
	Abstraction		≥26: Normal cognition
	Delayed recall		
	Orientation		
SAS	Anxiety symptoms	Contains 20 items (15 positive‑worded, 5 negative‑worded). Raw sum score multiplied by 1.25 yields the standard score.	<50: Normal
			50–59: Mild anxiety
			60–69: Moderate anxiety
			>69: Severe anxiety
SDS	Depressive symptoms	Contains 20 items (10 positive‑worded, 10 negative‑worded). Raw sum score multiplied by 1.25 yields the standard score.	<53: Normal
			53–62: Mild depression
			63–72: Moderate depression
			>72: Severe depression

MoCA: Montreal Cognitive Assessment; SAS:Self-Rating Anxiety Scale; SDS:Self-Rating Depression Scale.

### Statistical analyses

Categorical variables were expressed as frequency (percentage), whereas continuous variables were presented as median (interquartile range). The chi-square test was used to compare differences across groups for categorical variables. The independent samples t-test or Mann–Whitney test was adopted according to data distribution. Univariate and multivariate logistic regression analyses were performed to explore factors associated with possible cognitive impairment, anxiety, and depression. All statistical analyses were conducted using SPSS version 27.0 (IBM SPSS Inc., Chicago, USA). *p* < 0.05 was considered statistically significant.

This study was approved by the Institutional Review Board of The First Hospital of China Medical University on June 26, 2023 (Approval No. 2023-338-2). Written informed consent was obtained from all enrolled participants prior to data collection.

## Results

### Baseline characteristics

Of the 836 participants, 708 preoperative patients with DTC were finally included in the study. In total, 128 patients were excluded, including 82 with a postoperative pathological diagnosis other than DTC, 14 with a history of anxiety or depression, 26 with a history of thyroid surgery, and 6 with incomplete data. The screening process was shown in Figure 1.

**Figure 1:**
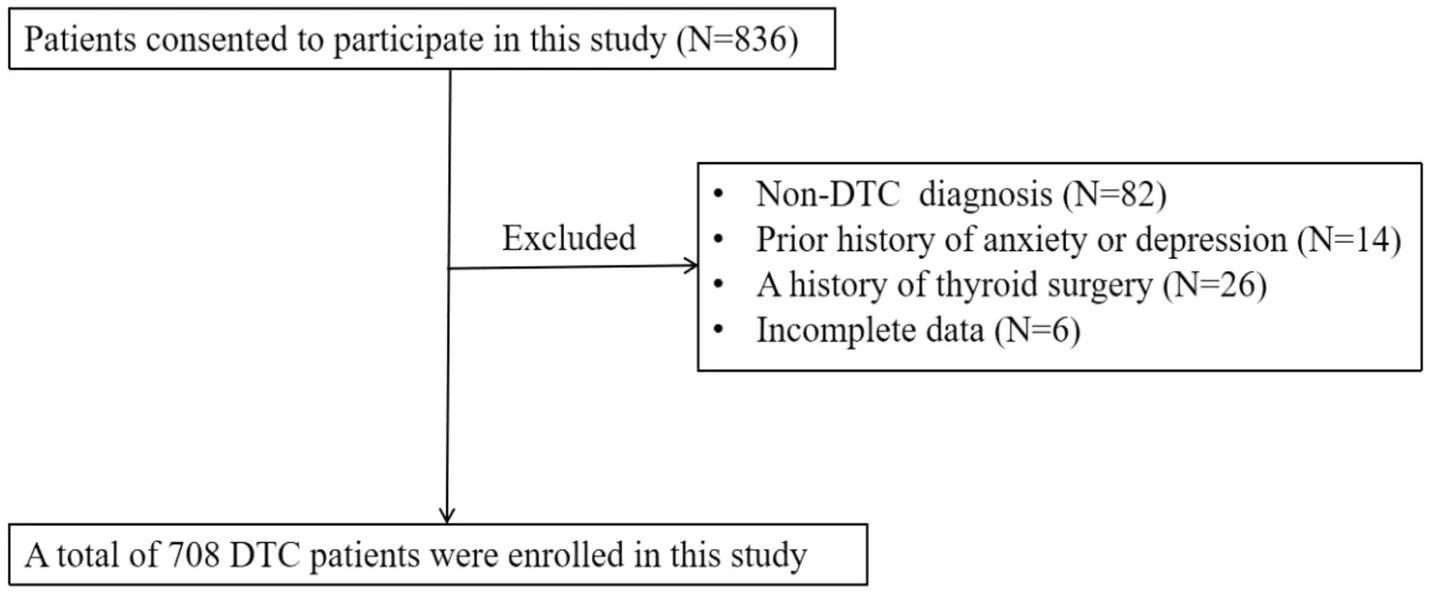
Flow chart of the study participant selection.

All included patients were newly diagnosed with DTC and were scheduled for thyroid surgery according to the 2015 ATA guidelines (Haugen et al., 2016). In total, 708 patients were enrolled in this study. The median age was 43 years (IQR, 36–50 years); most participants were ≤60 years old (682, 96.3%), and only 26 (3.7%) were over 60 years. The cohort was predominantly female (550, 77.7%). Among the 550 female participants, 136 were postmenopausal, whereas 414 were premenopausal. Overall, 7.2% (51/708) of patients presented anxiety symptoms, and 20.3% (144/708) had depressive symptoms. Possible cognitive impairment was observed in 62.7% (444/708) of participants. Thyroid function was available for 607 patients, with median FT3 of 4.5 pmol/L, FT4 of 13.2 pmol/L, and TSH of 1.5 uIU/ml. Baseline demographic and clinical characteristics were shown in Table 2.

**Table 2:** DTC patient’s demographic and clinical characteristics at baseline.

Variables	Participants (*n*,%/Median, IQR)
Age	43 (36, 50)
Age	
≤60	682 (96.3%)
>60	26 (3.7%)
Gender	
Female	550 (77.7%)
Male	158 (22.3%)
Menopausal status#	
Yes	136
No	414
BMI	
<18	23 (3.2%)
18–25	364 (51.4%)
25–30	246 (34.7%)
>30	75 (10.6%)
Comorbid conditions	
No	560 (79.1%)
Yes	148 (20.9%)
Education Levels	
Primary school	35 (4.9%)
Middle school	195 (27.5%)
High school	118 (16.7%)
University or above	360 (50.8%)
Marital status	
Unmarried	65 (9.2%)
Married	605 (85.5%)
Widowed	14 (2.0%)
Divorced	24 (3.3%)
Work status	
No work	215 (30.3%)
In work	392 (55.4%)
Retirement	101 (14.3%)
Income status (RMB)	
<2000	122 (17.2%)
2000–4,000	233 (32.9%)
4,000–6,000	168 (23.7%)
>6,000	185 (26.1%)
Residence	
Rural	171 (24.2%)
City	537 (75.8%)
Anxiety	
No	657 (92.8%)
Yes	51 (7.2%)
Mild	45 (6.4%)
Moderate	5 (0.7%)
Severe	1 (0.1%)
Depression	
No	564 (79.7%)
Yes	144 (20.3%)
Mild	126 (17.8%)
Moderate	18 (2.5%)
Severe	0
Cognitive impairment	
No	264 (37.3%)
Yes	444 (62.7%)
Thyroid hormone biomarkers	
FT3 (pmol/L)*	4.5 (4.3-4.9)
FT4 (pmol/L)*	13.2 (12.2-14.3)
TSH (uIU/ml)*	1.5 (1.0-2.1)

*Analysis performed among 607 patients due to incomplete data collection for the remaining participants. # Analyses regarding menopausal status (post- vs pre-menopausal) were restricted to 550 female participants. Comorbid conditions were defined as pre-existing chronic illnesses prior to thyroid surgery, including hypertension, diabetes mellitus, and coronary heart disease. Median IQR: median and interquartile range. RMB: Renminbi (Chinese Yuan).

### Group comparisons of possible cognitive impairment, anxiety and depression

Group comparisons were conducted to examine factors related to possible cognitive impairment, anxiety, and depression, as shown in Table 3. Among female participants, postmenopausal status was significantly associated with possible cognitive impairment (*p* < 0.001) and anxiety symptoms (*p* = 0.027). Comorbid conditions, education levels, marital status, income status, and residence differed significantly across groups stratified by possible cognitive impairment status (all *p* < 0.05). Income status and residence showed significant differences between patients with and without depressive symptoms. No significant differences in thyroid hormone biomarkers were detected across groups.

**Table 3:** Results of the MoCA, SAS and SDS assessments.

Variables	MoCA			SAS			SDS		
	No cognitive impairment (*n*, %)	Cognitive impairment (*n*, %)	*p-*value	No anxiety (*n*, %)	Anxiety (*n*, %)	*p*-value	No depression (*n*, %)	Depression (*n*, %)	*p-*value
Total	264 (37.3%)	444 (62.7%)		657 (92.8%)	51 (7.2%)		564 (79.7%)	144 (20.3%)	
Gender			0.604			0.630			0.345
Female	215 (39.1%)	335 (60.9%)		509 (92.5%)	41 (7.5%)		434 (78.9%)	116 (21.1%)	
Male	49 (31.0%)	109 (69.0%)		148 (93.7%)	10 (6.3%)		130 (82.3%)	28 (17.7%)	
Menopausal status#	18 (13.2%)		<0.001			0.027			0.683
No	197 (47.6%)	217 (52.4%)		389 (94.0%)	25 (6.0%)		325 (78.5%)	89 (21.5%)	
Yes	18 (13.2%)	118 (86.8%)		120 (88.2%)	16 (11.8%)		109 (80.1%)	27 (19.9%)	
BMI			0.508			0.382			0.186
<18	10 (43.5%)	13 (56.5%)		23 (100%)	0		18 (78.3%)	5 (21.7%)	
18–25	143 (39.3%)	221 (60.7%)		335 (92.0%)	29 (8.0%)		280 (76.9%)	84 (23.1%)	
25–30	83 (33.7%)	163 (66.3%)		231 (93.9%)	15 (6.1%)		207 (84.1%)	39 (15.9%)	
>30	28 (37.3%)	47 (62.7%)		68 (90.7%)	7 (9.3%)		59 (78.7%)	16 (21.3%)	
Comorbid conditions			0.004			0.403			0.476
No	224 (40.0%)	336 (60.0%)		522 (93.2%)	38 (6.8%)		443 (79.1%)	117 (20.9%)	
Yes	40 (27.0%)	108 (73.0%)		135 (91.2%)	13 (8.8%)		121 (81.8%)	27 (18.2%)	
Education Levels			0.001			0.964			0.066
Primary school	2 (5.7%)	33 (94.3%)		33 (94.3%)	2 (5.7%)		27 (77.1%)	8 (22.9%)	
Middle school	44 (22.6%)	151 (77.4%)		182 (93.3%)	13 (6.7%)		148 (75.9%)	47 (24.1%)	
High school	41 (34.7%)	77 (65.3%)		109 (92.4%)	9 (7.6%)		88 (74.6%)	30 (25.4%)	
University or above	177 (49.2%)	183 (50.8%)		333 (92.5%)	27 (7.5%)		301 (83.6%)	59 (16.4%)	
Marital status			0.001			0.771			0.192
Unmarried	39 (60.0%)	26 (40.0%)		62 (95.4%)	3 (4.6%)		53 (81.5%)	12 (18.5%)	
Married	212 (35.0%)	393 (65.0%)		559 (92.4%)	46 (7.6%)		483 (79.8%)	122 (20.2%)	
Widowed	3 (21.4%)	11 (78.6%)		13 (92.9%)	1 (7.1%)		8 (57.1%)	6 (42.9%)	
Divorced	10 (41.7%)	14 (58.3%)		23 (95.8%)	1 (4.2%)		20 (83.3%)	4 (16.7%)	
Work status			0.054			0.393			0.971
No work	66 (30.7%)	149 (69.3%)		198 (92.1%)	17 (7.9%)		172 (80.0%)	43 (20.0%)	
In work	156 (39.8%)	236 (60.2%)		362 (92.3%)	30 (7.7%)		311 (79.3%)	81 (20.7%)	
Retirement	42 (41.6%)	59 (58.4%)		97 (96.0%)	4 (4.0%)		81 (80.2%)	20 (19.8%)	
Income status (RMB)			<0.001			0.904			0.035
<2000	23 (18.9%)	99 (81.1%)		115 (94.3%)	7 (5.7%)		89 (73.0%)	33 (27.0%)	
2000–4,000	78 (33.5%)	155 (66.5%)		215 (92.3%)	18 (7.7%)		179 (76.8%)	54 (23.2%)	
4,000–6,000	70 (41.7%)	98 (58.3%)		155 (92.3%)	13 (7.7%)		139 (82.7%)	29 (17.3%)	
>6,000	93 (50.3%)	92 (49.7%)		172 (93.0%)	13 (7.0%)		157 (84.9%)	28 (15.1%)	
Residence			<0.001			0.914			0.026
Rural	36 (21.1%)	135 (78.9%)		159 (93.0%)	12 (7.0%)		126 (73.7%)	45 (26.3%)	
City	228 (42.5%)	309 (57.5%)		498 (92.7%)	39 (7.3%)		438 (81.6%)	99 (18.4%)	
FNA			0.681			0.134			0.603
No	101 (38.3%)	163 (61.7%)		240 (90.9%)	24 (9.1%)		213 (80.7%)	51 (19.3%)	
Yes	163 (36.7%)	281 (63.3%)		417 (93.9%)	27 (6.1%)		351 (79.1%)	93 (20.9%)	
FT3*	4.6 (4.3-5.0)	4.6 (4.3–5.0)	0.732	4.6 (4.3–4.9)	4.5 (4.2–4.9)	0.630	4.6 (4.3–4.9)	4.5 (4.2–4.9)	0.438
FT4*	13.2 (12.2-14.3)	13.2 (12.2–14.3)	0.757	13.2 (12.2–14.3)	13.0 (12.2–14.2)	0.993	13.2 (12.2–14.3)	13.22 (12.3–14.3)	0.631
TSH*	1.5 (1.0–2.3)	1.5 (1.0–2.3)	0.406	1.4 (1.0–2.1)	1.4 (1.0–2.4)	0.595	1.4 (1.0–2.1)	1.5 (1.0–2.2)	0.382

#Analyses for perimenopause-related outcomes (MoCA, SAS, SDS) were restricted to 550 female participants, as perimenopause status applies only to women. *Thyroid hormone data were available in 607 participants. Median IQR: median and interquartile range. RMB: Renminbi (Chinese Yuan). Comorbid conditions were defined as pre-existing chronic illnesses prior to thyroid surgery, including hypertension, diabetes mellitus, and coronary heart disease.

### Univariate and multivariate logistic regression analyses for possible cognitive impairment

Univariate and multivariate logistic regression analyses were performed to explore factors associated with possible cognitive impairment. Variables with *p* < 0.05 in the univariate logistic regression analyses and clinically relevant covariates were entered into multivariate logistic regression models. Two separate multivariate models were constructed. The first multivariate logistic regression model, which incorporated menopausal status, was fitted exclusively among female participants (*n* = 550; Table 4). The second model excluded menopausal status and was fitted in the overall study population (*n* = 708; Supplementary Table 1).

**Table 4:** Multivariate logistic regression analyses of factors associated with possible cognitive impairment among female participants (*n* = 550).

Characteristics	Univariate analysis			Multivariate analysis		
	*β*	OR 95%CI	*p-*value	*β*	OR 95%CI	*p-*value
Gender						
Female	Reference					
Male	−0.356`	0.700 (0.480–1.022)	0.065			
Menopausal status#						
Yes	Reference			Reference		
No	−1.784	0.168 (0.099–0.286)	<0.001	−0.940	0.390 (0.198–0.771)	0.007
Age	0.078	1.081 (1.062–1.101)	<0.001	0.043	1.044 (1.015–1.073)	0.003
BMI						
<18	Reference					
18–25	−0.256	0.774 (0.300–1.998)	0.597			
25–30	−0.083	0.921 (0.551–1.538)	0.752			
>30	0.157	1.170 (0.684–2.002)	0.567			
Comorbid conditions						
No	Reference			Reference		
Yes	0.993	0.556 (0.372–0.829)	0.004	−0.002	0.998 (0.573–1.738)	0.993
Education levels						
Primary school	Reference			Reference		
Middle school	2.770	15.959 (3.773–67.504)	<0.001	1.686	5.398 (1.150–25.342)	0.033
High school	1.200	3.319 (2.238–4.923)	<0.001	0.290	1.336 (0.786–2.271)	0.285
University or above	0.597	1.816 (1.180–2.797)	0.007	0.117	1.124 (0.648–1.950)	0.677
Marital status						
Unmarried	Reference					
Married	−0.742	0.476 (0.184–1.233)	0.126			
Widowed	0.281	1.324 (0.578–3.032)	0.507			
Divorced	0.963	2.619 (0.577–11.888)	0.212			
Work status						
No work	Reference			Reference		
In work	0.474	1.607 (0.984–2.625)	0.058	0.524	1.689 (0.896–3.186)	0.105
Retirement	0.074	1.077 (0.691–1.679)	0.744	0.111	1.117 (0.623–2.005)	0.710
Income status (RMB)						
<2000	Reference			Reference		
2000–4,000	1.470	4.351 (2.542–7.448)	<0.001	0.554	1.741 (0.859–3.529)	0.124
4,000–6,000	0.698	2.009 (1.351–2.986)	<0.001	0.199	1.221 (0.733–2.032)	0.443
>6,000	0.347	1.415 (0.929–2.156)	0.106	0.183	1.201 (0.705–2.046)	0.500
Residence						
Rural	Reference			Reference		
City	1.018	2.767 (1.845–4.151)	<0.001	0.493	1.638 (0.951–2.821)	0.075
FNA						
No	Reference					
Yes	−0.066	0.936 (0.684–1.282)	0.681			
FT3*	−0.051	0.950 (0.722–1.251)	0.717			
FT4*	−0.015	0.986 (0.894–1.086)	0.769			
TSH*	−0.018	0.982 (0.846–1.140)	0.811			

Menopausal status, age, comorbid conditions, educational levels, work status, income status, and place of residence were included in the multivariate regression analysis. # Analyses regarding menopausal status (post- vs pre-menopausal) were restricted to 550 female participants. *Thyroid hormone data were available in 607 participants. Comorbid conditions were defined as pre-existing chronic illnesses prior to thyroid surgery, including hypertension, diabetes mellitus, and coronary heart disease. RMB: Renminbi (Chinese Yuan).

When menopausal status was incorporated into the multivariate regression analysis, postmenopausal status (*p =* 0.007), age (*p* = 0.003), and educational level (*p* < 0.05) were independently associated with possible cognitive impairment (Table 4). When menopausal status was not incorporated into the multivariate regression analysis, age (*p* < 0.001), work status (*p* = 0.049), and residence (*p* = 0.007) were related to possible cognitive impairment (Supplementary Table 1).

### Univariate and multivariate logistic regression analyses for anxiety and depression

We used logistic regression analyses to explore factors correlated with anxiety and depressive symptoms. In univariate analyses, both menopausal status (*p* = 0.030) and TSH levels (*p* = 0.014) were significantly correlated with anxiety (Table 5). For depressive symptoms, multivariate regression analysis demonstrated that income status (*p* < 0.05) was independently associated with these symptoms (Table 6).

**Table 5:** Univariate logistic regression analyses of factors associated with anxiety in the overall cohort (*n* = 708)

Characteristics	Univariate analysis		
	*β*	OR 95%CI	*p-*value
Gender			
Female	Reference		
Male	0.176	1.192 (0.583–2.437)	0.630
Menopausal status#			
Yes	Reference		
No	−0.730	0.482 (0.249–0.933)	0.030
Age	0.017	1.017 (0.989–1.046)	0.240
BMI			
<18	Reference		
18–25	−18.929	NE	0.998
25–30	−0.173	0.841 (0.354–1.998)	0.695
>30	−0.461	0.631 (0.247–1.610)	0.335
Comorbid conditions			
No	Reference		
Yes	−0.28	0.756 (0.392–1.459)	0.404
Education levels			
Primary school	Reference		
Middle school	−0.291	0.747 (0.170–3.284)	0.700
High school	−0.127	0.881 (0.444–1.749)	0.717
University or above	0.018	1.018 (0.465–2.232)	0.964
Marital status			
Unmarried	Reference		
Married	0.107	1.113 (0.110–11.248)	0.928
Widowed	0.638	1.893 (0.250–14.333)	0.537
Divorced	0.571	1.769 (0.102–30.709)	0.695
Work status			
No work	Reference		
In work	0.733	2.082 (0.682–6.355)	0.198
Retirement	0.698	2.010 (0.691–5.842)	0.200
Income status (RMB)			
<2,000	Reference		
2,000–4,000	−0.216	0.805 (0.312–2.080)	0.655
4,000–6,000	0.102	1.108 (0.528–2.324)	0.787
>6,000	0.104	1.110 (0.499–2.467)	0.798
Residence			
Rural	Reference		
City	−0.037	0.964 (0.493–1.886)	0.914
FNA			
No	Reference		
Yes	0.435	1.544 (0.871–2.737)	0.137
FT3*	−0.145	0.865 (0.508–1.473)	0.594
FT4*	0.029	1.029 (0.864–1.226)	0.746
TSH*	0.267	1.306 (1.054–1.618)	0.014

# Analyses regarding menopausal status (post- vs. pre-menopausal) were restricted to 550 female participants. *Thyroid hormone data were available in 607 participants. Comorbid conditions were defined as pre-existing chronic illnesses prior to thyroid surgery, including hypertension, diabetes mellitus, and coronary heart disease. NE: not estimable, NE indicates that the OR and 95% CI for BMI 18–25 could not be reliably estimated because of sparse cell counts and unstable parameter estimation. RMB: Renminbi (Chinese Yuan).

**Table 6:** Multivariate logistic regression analyses of factors associated with depressive symptoms in the overall cohort (*n* = 708).

Characteristics	Univariate analysis			Multivariate analysis		
	*β*	OR 95%CI	*p-*value	*β*	OR 95%CI	*p-*value
Gender						
Female	Reference					
Male	0.216	1.2421 (0.786–1.960)	0.354			
Menopausal status#						
Yes	Reference					
No	0.100	1.106 (0.683–1.791)	0.683			
Age	0.006	1.006 (0.988–1.025)	0.500			
BMI						
<18	Reference					
18–25	0.024	1.024 (0.329–3.185)	0.967			
25–30	0.101	1.106 (0.605–2.024)	0.743			
>30	−0.364	0.695 (0.363–1.331)	0.272			
Comorbid conditions						
No	Reference					
Yes	0.169	1.184 (0.744–1.883)	0.477			
Education levels						
Primary school	Reference			Reference		
Middle school	0.413	1.512 (0.655–3.490)	0.333	−0.030	0.970 (0.386–2.443)	0.949
High school	0.483	1.620 (1.053–2.492)	0.028	0.185	1.204 (0.728–1.991)	0.471
University or above	0.553	1.739 (1.055–2.866)	0.030	0.433	1.542 (0.920–2.582)	0.100
Marital status						
Unmarried	Reference					
Married	0.124	1.132 (0.327–3.924)	0.845			
Widowed	0.233	1.263 (0.424–3.763)	0.675			
Divorced	1.322	3.750 (0.830–16.935)	0.086			
Work status						
No work	Reference					
In work	0.012	1.012 (0.560–1.831)	0.967			
Retirement	0.053	1.055 (0.610–1.823)	0.848			
Income status (RMB)						
<2000	Reference			Reference		
2000–4,000	0.732	2.079 (1.180–3.664)	0.011	0.732	2.079 (1.180–3.664)	0.011
4,000–6,000	0.526	1.692 (1.022–2.801)	0.041	0.526	1.692 (1.022–2.801)	0.041
>6,000	0.157	1.170 (0.663–2.063)	0.588	0.157	1.170 (0.663–2.063)	0.588
Residence						
City	Reference					
Rural	0.457	1.580 (1.055–2.367)	0.027			
FNA						
No	Reference					
Yes	−0.101	0.904 (0.617–1.324)	0.603			
FT3*	−0.213	0.808 (0.569–1.148)	0.234			
FT4*	0.012	1.012 (0.901-1.137)	0.838			
TSH*	0.059	1.060 (0.892-1.261)	0.507			

We incorporated education levels, income status, residence into the multivariate regression model. Comorbid conditions were defined as pre-existing chronic illnesses prior to thyroid surgery, including hypertension, diabetes mellitus, and coronary heart disease. *Thyroid hormone data were available in 607 participants. # Analyses regarding menopausal status (post- vs pre-menopausal) were restricted to 550 female participants. RMB: Renminbi (Chinese Yuan).

## Discussion

To our knowledge, this is the first large-scale study to explore baseline possible cognitive impairment and emotional disturbances in China. This study is of great significance, since it not only adds to existing research on cancer-related cognitive impairment, but also enhances clinicians’ awareness of the mental health of patients newly diagnosed with DTC before treatment. In this study, the rates of possible cognitive impairment, anxiety, and depression were 62.7, 7.2, and 20.3%, respectively, which were significantly higher than those in the general population (Lu et al., 2021; Huang et al., 2019). A cancer diagnosis may trigger anxiety, fear, and other negative emotions, which could in turn interfere with cognitive performance and impair quality of life (Pitt et al., 2021). Therefore, it is valuable to comprehensively identify the factors related to possible cognitive impairment and emotional disorders among DTC patients.

### Factors affecting cognitive function in DTC

Cumulative evidence has demonstrated that DTC patients experience impairment in multiple cognitive domains, such as memory, attention, executive function, and processing speed, during treatment (Wefel et al., 2015). Consistent with previous findings (Larrabee et al., 2023), our study identified a high prevalence (62.7%) of possible cognitive impairment among preoperative DTC patients. In addition, the MoCA scale is highly sensitive in screening for mild cognitive impairment and allows effective detection of preoperative possible cognitive impairment, further underscoring its clinical value. The development of possible cognitive impairment is multifactorial. It is closely linked to hippocampal atrophy, morphological deformation, and altered functional connectivity (Peukert et al., 2020). In addition, changes in cytokine and oxidative stress levels in the tumor microenvironment also play a role (Ren et al., 2017). Genetic predisposition and protein deposition further exacerbate cognitive deficits. For example, APOEε4 and amyloid substance deposition impair patients’ attention, learning ability, and cognitive processing speed (Reinvang et al., 2013). Moreover, fluctuations in thyroid hormone levels can alter hippocampal hormonal responsiveness, which in turn induces a series of psychological and cognitive alterations in DTC patients (Jurado-Flores et al., 2022; Eslami-Amirabadi and Sajjadi, 2021; Bavarsad et al., 2019).

In multivariate analysis, perimenopausal status, age, educational level, work status, and residence were independently associated with possible cognitive impairment. Multiple studies have explored the biological mechanisms underlying these associations. Estrogen contributes to neuroprotective effects and supports neuronal development, connectivity, and plasticity. During the perimenopausal transition, declining ovarian function leads to reduced estrogen levels, which may impair cognitive performance in women (Metcalf et al., 2023). Aging is accompanied by increased neuronal death and reduced synaptic connections. Furthermore, the incidence of cardiovascular and cerebrovascular diseases such as diabetes and hypertension rises with advancing age, accompanied by alterations in cerebral blood flow, which further contribute to cognitive deficits. The hippocampus serves as the core structure for learning, memory, and cognitive functions, and it is highly vulnerable to hypoxic ischemic injury (Johnson, 2023).

Education levels reflect knowledge reserves and helps strengthen brain network connectivity, preserves cognitive function, maintaining normal cognitive function, and resisting brain aging (Du et al., 2023). A higher educational level is also associated with enhanced information processing, problem solving, and decision-making abilities (Jefferson et al., 2011). Patients with higher educational levels tend to have better understanding and awareness regarding the diagnosis, treatment, and prognosis of DTC. In contrast, individuals with lower educational levels are more likely to develop adverse cognitive perceptions.

Individuals living in rural areas often have relatively lower income, lower education levels, and insufficient access to health related information, which may contribute to increased psychological distress (Du et al., 2023). Therefore, adequate attention and informational support should be offered to patients with these risk factors before treatment to avoid further cognitive deterioration.

### Factors affecting anxiety and depression in DTC

A cancer diagnosis and concerns regarding subsequent treatment may increase anxiety, depression, and fear among patients with DTC (Pitt et al., 2021). In a study conducted in 2022, the prevalence of anxiety in patients with thyroid cancer was as high as 37.6%, particularly in women with elevated BMI, recurrent laryngeal nerve injury, or hypoparathyroidism (Noto et al., 2022). This proportion is substantially higher than the anxiety prevalence observed in our cohort (7.2%). This discrepancy may be partially explained by our exclusion of 14 participants with prior anxiety or depression. In addition, differences in recruitment settings, assessment instruments, timing of evaluation, and patient demographic characteristics may also account for divergent findings. Our study enrolled preoperative patients, whereas the study by Noto et al. included postoperative individuals. Postoperative patients tend to pay more attention to treatment related complications, such as recurrent laryngeal nerve injury, hypoparathyroidism, and scar-related problems (Campagnoli et al., 2023).

Postoperative RAI treatment, TSH suppressive therapy, and potential thyroid dysfunction (including hyperthyroidism (Tagay et al., 2005), hypothyroidism (Tagay et al., 2006)) during treatment can also increase the patient’s anxiety levels. Economic burden and anticipation of final pathological results also contribute to psychological distress (Mongelli et al., 2020; Yang and Xu, 2022). In our study, the prevalence of anxiety was lower but still significantly higher than that in general populations (Huang et al., 2019). In univariable analysis, postmenopausal status was associated with greater odds of anxiety among women. This trend merits consideration, as it may be partly explained by hormonal fluctuations and menopausal related symptoms in postmenopausal females (Campagnoli et al., 2023). In the Chinese context, women entering retirement commonly experience shifts in lifestyle and living circumstances. Therefore, more attention should be paid to patients in perimenopausal period.

In this study, up to 20.3% of the patients reported depressive symptoms, with income level identified as an independent correlate. This prevalence was higher than that reported in a previous study (Noto et al., 2022). Several factors may account for this discrepancy. Preoperative patients often face financial pressure, which may represent an important contributor to depressive symptoms (Srivastava et al., 2021). Furthermore, long-term subsequent cancer treatment may also affect the future economic status of the family, and this concern is more prominent among patients with lower income levels (Bresner et al., 2015).

In addition, the income level is closely related to knowledge reserves (Herrera-Escobar et al., 2019). Individuals with higher income level are more likely to achieve higher educational levels, which allows them to acquire more comprehensive knowledge about their disease. This may alleviate concerns regarding surgical risks, postoperative recovery, and potential complications (e.g., surgical scars).

### Limitations

This study has several limitations. As a cross-sectional investigation, it cannot establish causality. We could not distinguish whether possible cognitive impairment existed prior to cancer diagnosis or resulted from diagnosis related stress; accordingly, variables identified in regression analyses represent correlates rather than causal risk factors.

Recruitment from a single tertiary hospital may limit generalizability, as enrolled patients tended to have more complex thyroid conditions and higher comorbidity burdens compared with individuals in community. Voluntary participation might introduce selection bias, given potential psychological differences between responders and non-responders. Information bias cannot be fully excluded because psychological and cognitive outcomes relied on self-reported questionnaires.

Several key variables were unavailable before surgery, including detailed DTC pathological subtype information (e.g., follicular variant and tall-cell variant of papillary thyroid carcinoma), planned surgical approach, and intended RAI therapy. Residual confounding may exist due to unmeasured factors such as social support, the interval between diagnosis and assessment, family history of thyroid disease, and reasons for clinical consultation. Furthermore, formal adjustment for multiple comparisons was not conducted for these exploratory analyses, which may increase the risk of Type I error. Future prospective studies incorporating these unmeasured variables will help better characterize psychological outcomes among DTC patients.

## Conclusion

Clinicians should pay attention to the psychological and cognitive status of patients newly diagnosed with DTC before treatment. Our findings suggest that postmenopausal status, age and educational levels are independent correlates of possible cognitive impairment in women, whereas age, work status, and residence are independent correlates in the overall cohort. TSH levels are associated with anxiety, and income status is an independent correlate of depressive symptoms. Given the cross-sectional design, causal inferences cannot be drawn from the present study. Future prospective studies are needed to validate these associations and clarify relevant mechanisms.

## Data Availability

The raw data supporting the conclusions of this article will be made available by the authors, without undue reservation.
