# Supplementary material for: Cognitive function, anxiety, and depression in patients newly diagnosed with thyroid cancer in China
Source: Front Psychol. 2026 Sep 21;17:1908212. doi: 10.3389/fpsyg.2026.1908212 (PMC13635319; doi:10.3389/fpsyg.2026.1908212)
Supplement: Supplementary file 1 [file Table_1.pdf]

Supplementary Table 1:Multivariate logistic regression analyses of factors associated with possible cognitive impairment in the overall cohort(n=708)

		Univariate analysis			Multivariate analysis		
Characteristics		β	OR 95%CI	*p*-value	β	OR 95%CI	*p*-value
Gender							
	Female	Reference					
	Male	-0.356	0.700(0.480-1.022)	0.065			
Menopausal status							
	yes	Reference					
	No	-1.784	0.168(0.099-0.286)	<0.001			
Age		0.078	1.081(1.062-1.101)	<0.001	0.065	1.067(1.046-1.089)	<0.001
BMI							
	<18	Reference					

	18-25		-0.256	0.774(0.300-1.998)	0.597				
	25-30		-0.083	0.921(0.551-1.538)	0.752				
	>30		0.157	1.170(0.684-2.002)	0.567				
Comorbid conditions									
	No	Reference				Reference			
	Yes		0.993	0.556(0.372-0.829)	0.004		-0.218	0.804(0.516-1.253)	0.335
Education Levels									
	Primary school	Reference				Reference			
	Middle school		2.770	15.959(3.773-67.504)	<0.001		1.477	4.378(0.962-19.924)	0.056
	High school		1.200	3.319(2.238-4.923)	<0.001		0.375	1.455(0.899-2.356)	0.127
	University or above		0.597	1.816(1.180-2.797)	0.007		0.018	1.018(0.628-1.651)	0.941
Marital status									
	Unmarried	Reference							

	Married		-0.742	0.476(0.184-1.233)	0.126				
	Widowed		0.281	1.324(0.578-3.032)	0.507				
	Divorced		0.963	2.619(0.577-11.888)	0.212				
Work status									
	No work	Reference				Reference			
	In work		0.474	1.607(0.984-2.625)	0.058		0.547	1.728(1.002-2.980)	0.049
	Retirement		0.074	1.077(0.691-1.679)	0.744		0.177	1.194(0.725-1.965)	0.487
Income status(RMB)									
	<2000	Reference				Reference			
	2000-4000		1.470	4.351(2.542-7.448)	<0.001		0.504	1.655(0.866-3.162)	0.127
	4000-6000		0.698	2.009(1.351-2.986)	<0.001		0.241	1.272(0.818-1.978)	0.285
	>6000		0.347	1.415(0.929-2.156)	0.106		0.174	1.190(0.756-1.872)	0.452
Residence									

	City	Reference			Reference		
	Rural	1.018	2.767(1.845-4.151)	<0.001	0.684	1.982(1.204-3.263)	0.007
FNA							
	No	Reference					
	Yes	-0.066	0.936(0.684-1.282)	0.681			
FT3*		-0.051	0.950(0.722-1.251)	0.717			
FT4*		-0.015	0.986(0.894-1.086)	0.769			
TSH*		-0.018	0.982(0.846-1.140)	0.811			

Age, comorbid conditions, educational level, work status, income status, and place of residence were included in the multivariate regression analysis.

*Thyroid hormone data were available in 607 participants。

RMB:Renminbi (Chinese Yuan)
